# Mid-upper arm circumference predicts treatment outcomes in paediatric TB: a cohort study

**DOI:** 10.5588/ijtldopen.26.0040

**Published:** 2026-05-11

**Authors:** D. Van Aartsen, C. Cintron, S. Greenfield, M. Justine, E. Mduma, S. Mpagama, E.R. Houpt, M.R. Dauphinais, P. Sinha, S.K. Heysell, J.M. McDermid, T.A. Thomas

**Affiliations:** 1 Division of Infectious Diseases, Department of Medicine, Rush University Medical Center, Chicago, IL, USA;; 2 Division of Infectious Diseases and International Health, University of Virginia, Charlottesville, VA, USA;; 3 Department of Medicine, Boston Medical Center, Boston, MA, USA;; 4 Section of Infectious Diseases, Boston University Chobanin & Avedisian School of Medicine, Boston, MA, USA;; 5 Department of Epidemiology, Brown University, Providence, RI, USA;; 6 Center for Global Health Equity, University of Virginia, Charlottesville, VA, USA;; 7 Haydom Global Health Research Centre, Haydom Lutheran Hospital, Haydom, Tanzania;; 8 Kibong’oto Infectious Diseases Hospital, Sanya Juu, Tanzania.

**Keywords:** tuberculosis, anthropometry, nutrition, child TB, Tanzania

Dear Editor,

Low body mass index (BMI) is an important prognosticator for unfavourable treatment outcomes and mortality in adults with TB.^1,2^ Although prior studies have linked undernutrition to poor TB treatment outcomes in children, they have predominantly relied on weight-based indicators such as weight-for-height Z-score (WHZ) and weight-for-age Z-score (WAZ), which require accurate scales and height measurements. In contrast, mid-upper arm circumference (MUAC) offers a simpler, age-independent alternative, particularly in children aged 6–59 months.^3^ Through this single-site prospective cohort study, we aimed to characterise anthropometric changes during TB treatment among children in rural Tanzania, assess the correlation between MUAC and other anthropometric indicators, and test whether lower MUAC at TB diagnosis predicts poorer treatment outcomes.

Participants were part of a larger paediatric prospective cohort examining predictors of anti-TB pharmacokinetics in Haydom, Tanzania (Clinical Trial Registration: Diagnostics and Pharmacotherapy for Severe Forms of TB, ClinicalTrials.gov identifier NCT03559582).^4^ Located in Northern Tanzania, Haydom is a village comprising a population of subsistence farmers who are subject to seasonal food insecurity and childhood malnutrition.^5^ We included children under 5 years of age with probable or confirmed intra-thoracic drug-susceptible TB, as defined by the National Institutes of Health Consensus Case Definitions for TB research in children.^6^ First-line anti-TB treatment was initiated by the treating clinician as per Tanzanian guidelines.^7^ Anthropometry (height, weight, MUAC) was measured at TB treatment initiation and treatment weeks 2, 4, 8, and 24. All participants concomitantly diagnosed with severe acute malnutrition (SAM) were treated in the inpatient ward with therapeutic diets and micronutrients according to WHO guidelines.^8^ Participants with moderate acute malnutrition (MAM) were provided a nutritional regimen according to malnutrition severity while an inpatient, as per hospital policy, and caregivers were provided nutritional counselling upon discharge with regular outpatient monitoring.

MUAC Z-score, WAZ, height for age Z-score (HAZ), WHZ, and BMI Z-score were calculated according to WHO recommendations using the R package ‘anthro’.^9,10^ SAM was defined as MUAC < 11.5 cm or WHZ ≤ −3, MAM as MUAC 11.5–12.5 cm or WHZ > −3 and ≤−2, and at-risk for malnutrition as MUAC 12.5–13.5 cm or WHZ > −2 and ≤−1. Anthropometrics were measured up to 24 weeks, and outcomes at 48 weeks. Mortality was defined as death from any cause before 48 weeks. Treatment success was defined as completion of anti-TB therapy and complete symptom resolution at the time of week 48 assessment. Correlations of MUAC Z-score with WAZ, HAZ, WHZ, and BMI Z-score were performed by Pearson’s test. Logistic regression was used to compare baseline MUAC, WHZ, and WAZ, and severity of malnutrition at diagnosis to treatment outcomes and mortality. Multivariable regression was not conducted due to the small sample size and descriptive, exploratory nature of this analysis. Ethics approval was provided by the Tanzania National Institute for Medical Research and the University of Virginia.

A total of 41 children were enrolled (median age 1.6 years [interquartile range (IQR) 1.0–2.3], 51% female), of whom 27 (66%) had microbiologically confirmed TB and 10 (24.4%) had concurrent extra-pulmonary TB; none were living with HIV. Most children had impaired appetite (71%) and reported weight loss (93%) – see Table 1. The median baseline MUAC was 12.2 cm (IQR 11.0–13.3). At diagnosis, 17 (42%) met criteria for SAM, 7 (17%) for MAM, 8 (20%) were at risk, and 9 (22%) were not malnourished. MUAC was strongly correlated with MUAC Z-score (R = 0.95, *P* < 0.001), as well as with WAZ, WHZ, and BMIZ (R = 0.74, 0.69, 0.68, respectively; both *P* < 0.001).

**Table 1.** Baseline characteristics.

Characteristic	n = 41
Median age, years (IQR)	1.6 (1–2.3)
Female, n (%)	21 (51%)
Prior history of TB, n (%)	3 (7%)
Microbiologic confirmation, n (%)	27 (66%)
Median haemoglobin, mg/dL (IQR)	8.6 (7.8–10.8)
HIV positive, n (%)	0 (0%)
Extra-pulmonary TB disease, n (%)	10 (24%)
Pleural	1 (2%)
Lymphatic	8 (20%)
Pericardial	1 (2%)
Symptom duration, days	90 (60–180)
Fever > 1 week, n (%)	38 (93%)
Cough > 2 weeks, n (%)	40 (98%)
Sweats > 2 weeks, n (%)	35 (85%)
Haemoptysis, n (%)	4 (10%)
Impaired appetite > 2 weeks, n (%)	29 (71%)
Weight loss for > 2 weeks, n (%)	38 (93%)
Lethargy > 2 weeks, n (%)	38 (93%)

Presence of symptoms, symptom duration, and weight loss are caregiver-reported.

IQR = interquartile range.

**Figure 1.**
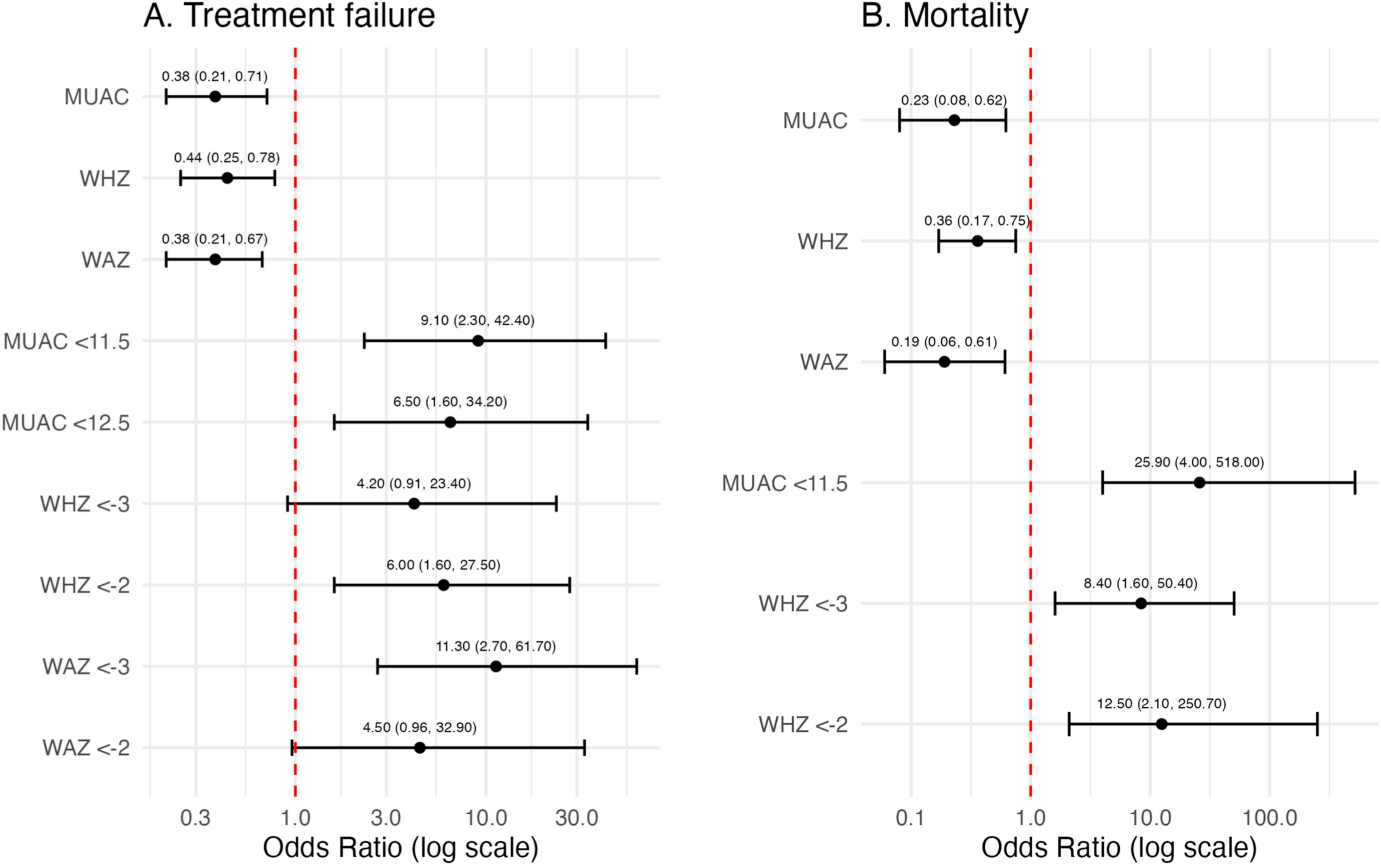
Forest plots of unadjusted odds ratios predicting (**A**) treatment failure and (**B**) mortality. MUAC = mid-upper arm circumference; WHZ = weight-for-height Z-score; WAZ = weight-for-age Z-score.

Twenty-four (59%) participants had treatment success, 16 (42%) experienced treatment failure, and 10 (24%) died. Among the 28 children with anthropometry data at week 24, 23 (82%) showed MUAC improvement, with a mean increase of 1.7 cm (14% from baseline; SD 8%–20%). Figure 1 shows unadjusted odds ratios (ORs) predicting treatment failure and mortality. Baseline MUAC, WAZ, and WHZ were each inversely associated with both treatment failure and mortality (all *P* < 0.01; Table 2). Baseline MUAC < 11.5 cm (SAM) predicted both treatment failure (OR 9.1, 95% confidence interval [CI]: 2.3–42.4) and mortality (OR 25.9, 95% CI: 4.0–518), while MUAC < 12.5 cm was associated with treatment failure (OR 6.5, 95% CI: 1.6–34.2). In contrast, WHZ ≤ −3 was associated with mortality (OR 8.4, 95% CI: 1.6–50.4) but not treatment failure, while WHZ ≤ −2 was associated with both outcomes. Notably, no deaths occurred among children with MUAC > 12.5 cm or WAZ > −3.

**Table 2.** Unadjusted logistic regression using baseline MUAC, WHZ, or WAZ to predict TB treatment failure or mortality, or using established WHO thresholds defining severe or moderate malnutrition (MUAC < 11.5 or <12.5 cm, WHZ < −3 or < −2, and WAZ < −3 or <−2).

Predictor	Treatment failure	Treatment failure	*P*	Mortality		*P*
	Estimate (SE)	Odds ratio (95% CI)		Estimate (SE)	Odds ratio (95% CI)	
MUAC	−0.957 (0.316)	0.38 (0.21, 0.71)	0.003	−1.475 (0.509)	0.23 (0.08, 0.62)	0.004
WHZ	−0.820 (0.291)	0.44 (0.25, 0.78)	0.005	−1.025 (0.374)	0.36 (0.17, 0.75)	0.006
WAZ	−0.968 (0.309)	0.38 (0.21, 0.67)	0.002	−1.666 (0.598)	0.19 (0.06, 0.61)	0.005
MUAC < 11.5	2.210 (0.732)	9.1 (2.3, 42.4)	0.003	3.253 (1.131)	25.9 (4.0, 518)	0.004
MUAC < 12.5	1.877 (0.759)	6.5 (1.6. 34.2)	0.01	19.230^**A**^ (2,608)	NA^B^	0.994
WHZ < −3	1.435 (0.805)	4.2 (0.91, 23.4)	0.07	2.133 (0.859)	8.4 (1.6, 50.4)	0.01
WHZ < −2	1.792 (0.722)	6.0 (1.6, 27.5)	0.01	2.539 (1.123)	12.5 (2.1, 250.7)	0.01
WAZ < −3	2.428 (0.779)	11.3 (2.7, 61.7)	0.002	19.471^**A**^ (24,040)	NA^B^	0.9
WAZ < −2	1.504 (0.863)	4.5 (0.96–32.9)	0.08	17.873^**A**^ (1,966)	NA^B^	0.9

No cases of mortality with MUAC > 12.5 cm or WAZ > −3 and odds ratios were not calculated.

MUAC = mid-upper arm circumference; WHZ = weight-for-height Z-score; WAZ = weight-for-age Z-score; CI = confidence interval.

A Estimates could not be properly calculated due to few cases of mortality in this group.

B Estimates could not be properly calculated due to zero cases of mortality in this group.

**Figure 2.**
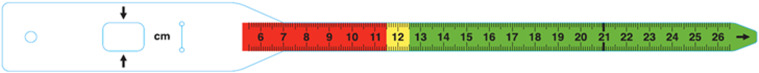
Mid-upper arm circumference measuring tape. Red: 0–11.5 cm (severe acute malnutrition), yellow: 11.5–12.5 cm (moderate acute malnutrition), green: from 12.5 cm (https://www.unicef.org/supply/mid-upper-arm-circumference-muac-measuring-tapes).

In this cohort of Tanzanian children with TB, we observed a high prevalence of malnutrition. Although WAZ measurements at treatment initiation identified children with malnutrition who were at greater risk of TB treatment failure or mortality, MUAC matched this measure while also offering a fixed, age- and sex-independent threshold that simplifies field implementation, making it uniquely practical in settings with high burdens of TB and malnutrition. Indeed, MUAC requires only a low-cost tape measure, which allows community caregivers to triage at-risk children for further evaluation and treatment, and monitor children’s nutritional status once discharged from the clinical setting.^11^ The built-in colour-coded risk categories further improve interpretability, potentially enhancing caregiver engagement, treatment adherence, and timely escalation of care (Figure 2).

Our study has some limitations. The findings are restricted to children under five, where the mortality burden is notably higher, and may not generalise to older children or adolescents. The cohort also had a higher prevalence of moderate and severe malnutrition than comparable studies, which may limit external validity; however, this enhances its relevance for impoverished or food-insecure settings.^12,13^ Additionally, anthropometric measures like MUAC cannot differentiate TB-related wasting from other causes of undernutrition.^14^ Finally, we did not compare MUAC with dietary intake or biochemical markers, which could further contextualise nutritional status.

In conclusion, this study suggests that MUAC is more than just a convenient field tool; it is a powerful predictor of treatment failure and mortality in young children with TB. Its simplicity, scalability, and prognostic strength make it uniquely suited for integration across the TB care continuum, from community-based screening to treatment monitoring.
